# Edible electronics: Current landscape and emerging pathways

**DOI:** 10.1557/s43578-025-01773-7

**Published:** 2026-01-20

**Authors:** Mario Caironi, Alessandro Luzio

**Affiliations:** Center for Nano Science and Technology, Istituto Italiano di Tecnologia, Milan, 20134 Italy

**Keywords:** Devices, Electrical properties, electronic materials, Energy storage, Environmental impact

## Abstract

Edible electronics was envisioned not long ago as a radical alternative approach to existing ingestible electronics, where all components are made of edible functional materials, which can be digested, metabolized, or simply excreted without threats for human health nor environmental concerns. In this contribution, we ought to draw a line and critically revise the most concrete developments achieved in this field since its recent conception. Our aim is to offer an overview of the most recent progress that allow reassessing the vision towards future edible systems. Challenges towards reliable and manufacturable edible electronic systems still withstand, and further critical specific aspects will have to be addressed, including regulations and acceptance. Nevertheless, the relevant scientific and technical progress has made opportunities in edible smart pills for gastrointestinal monitoring and food sensors for safe tracking and waste reduction more concrete. At the same time, broader opportunities emerge.

**Graphical abstract:**

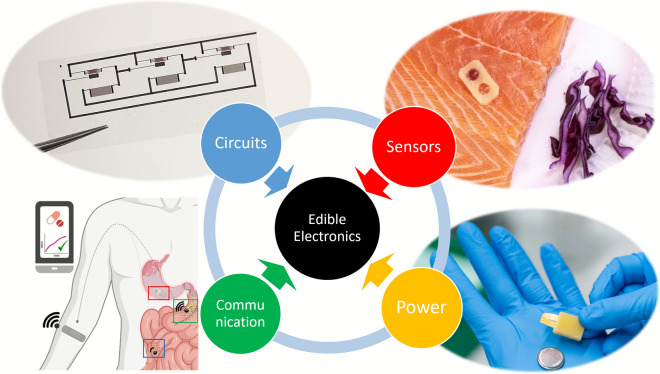

## Introduction

In 2020, with some collaborators, we published a review article about edible electronics. It meant to contribute to shaping a consistent vision of the field, which was inspired by a limited number of previous ground-breaking works and was still much in its infancy. In fact, examples in this direction were scarce and isolated, mostly focused on discrete, laboratory scale, electronic devices or components, resulting from efforts of only a few pioneering groups around the world. A strong contribution came from Irimia-Vladu, Glowacki, Sariciftci, Bauer, and co-workers in Linz, who introduced the first example of a mm-sized organic field-effect transistor (FET) on a gelatin capsule based on non-toxic materials [1]. While first appearing in Europe, examples of other edible components followed more often in the USA: the first current source composed entirely of edible materials found in common diets, [2] the first attempt at edible power supplies by Bettinger and co-workers at Carnagie-Mellon University; radio-frequency (RF) antennas based on edible metals patterned on silk proposed by Omenetto and co-workers at Tufts University; [3] food-based supercapacitors by Jiang and co-workers at Arizona State University [4]. The work of in het Panhuis and collaborators in Australia directly explored the 3D printing of food, namely commercial breakfast spreads from yeast extracts, for circuits interconnects, [5] as well as gelatin/gellan gum hydrogels for pressure sensors aimed at monitoring gut motility disorders [6].

This set of works, combined with a continuously increasing interest in gut health [7–9] and the necessity for sustainable technologies characterized by a drastically reduced life-cycle impact on the environment, [10] were the base that inspired the ERC Consolidator Project “Electronic Food: enabling edible electronic systems for biomedical and food monitoring applications” (ELFO), which engaged us in the last 5 years. A preliminary work by Bonacchini [11], in collaboration with Lanzani, was particularly relevant in shaping ELFO vision: the use of temporary tattoo paper, previously adopted in the context of ultra-conformable electronics, [12, 13] was proposed as a platform for the integration of printed organic electronics onto food and pharmaceutical capsules. ELFO, which ran from September 2020 until August 2025, was devised to enable a new generation of edible electronic systems by exploiting inherent electronic properties of food and food derivatives, suitably combined with edible, mostly carbon-based, synthetic materials. The vision was to develop advanced biomedical devices for prolonged monitoring of the health status within the gastrointestinal (GI) tract. Such electronics, being inherently edible, was suggested also for food labeling purposes, serving public health and offering safe and sustainable electronic tools against counterfeiting. ELFO planned to take a radical shift from ingestible electronic systems, sophisticated tools that, starting from the invention of the transistor in the 50s, [14] have been developed to provide real-time information from the inside of the GI tract.

Edible, safe-to-ingest devices mostly made of food, food derivatives, and approved additives offered an exciting research opportunity, with a fascinating long-term vision. Yet, challenges were enormous, starting from the fact that giving up silicon electronics for miniaturized systems that can fit into an ingestible object is by itself a potential road-blocker for many applications requiring highly reliable and highly performing electronics to meet existing standards. At the same time, the path of creating new standards for an alternative technology poses critical risks. Additionally, only a limited number of edible functional materials are candidates for re-building from scratch, one layer and one interface at a time, microelectronic devices with acceptable performances and durability [15, 16], and necessarily compatible with mass production at low cost to meet the huge volumes required for daily monitoring applications.

Thus, the question whether such an appealing scenario would be feasible at all. Here, we aim at a critical assessment of the most recent progress in edible electronics by focusing on advances in electronic components and system integration efforts, which we take as a tangible measure of the maturity of the field, and discuss whether such vision still holds and whether edible electronics is moving from an exotic, curiosity-driven research field to viable applications.

## What do we actually mean by edible electronics?

It is important to define in the first place what are the boundaries of edible electronics. We consider under this terminology all electronics that are realized only with functional materials, i.e., semiconductors, conductors, and insulators, that are safe to be ingested at the dose of use. The latter implies that the ingestion of a specific device does not exceed safety limits for topical and chronic consumption of the constituent materials set by international agencies, such as the European Food Safety Authority (EFSA) and the Food and Drugs Administration (FDA) in US. In fact, any substance, even a nutrient, has maximum topical and chronic levels of intake, for which it can be considered safe. In particular, in the case of materials that may provoke undesired effects for excessive consumption, agencies have defined acceptable daily intake (ADI) limits. In the field of edible electronics, it is therefore necessary to produce list of materials, as a sort of list of ingredients, with their specific amounts per device/system to be ingested, and to verify that the prospected use, albeit an estimation for an application in the long term, is at least compatible with the respect to ADI values. While we make explicit reference to the constituent, isolated materials, we highlight how the toxicological profile of the final complete device would in any case need to be reassessed, as interactions among materials could alter the way they are absorbed within the body [17].

The resulting device would follow the same fate of food after performing the intended function, being reduced to smaller parts along the GI tract, partly digested and possibly metabolized, and partly safely excreted. Sometimes literature could refer to the very same field with “food-based electronics” [18]. We expect that synonyms could also be used in the future or in less formal communications, such as “eatable electronics” or “comestible electronics.” Examples of edible electronic materials are present in food itself (e.g., marmite as an ionic conductor [5], carotenoids in vegetables as semiconductors [1, 19], polysaccharides and proteins as substrates, gelling agents for electrolytes and structural materials, [18, 20–26] corn zein as a glue [27, 28]), food and dietary supplements (e.g., redox molecules such as riboflavin and quercetin [29], activated carbon as conductive filler [28, 30, 31]), food additives (e.g., various salts, shellac, and chitosan as insulators, [32–36] ethylcellulose as substrate [26, 35, 36]), including food garnishments (e.g., 24 karat edible gold leaves [4, 18, 29, 37–39]). At the same time, within edible electronics, synthetic materials that are nontoxic upon ingestion may be adopted. In this case, suitable toxicological studies should be performed to prove edibility [40]. Alternatively, one can resort to materials, for example among those approved in cosmetics, that, despite not being approved for consumption, are daily ingested without registered adverse reactions. As a concrete example, recently we evidenced that semiconducting copper phthalocyanine (CuPc), a blue pigment used in toothpastes as a whitening agent, is among such definition, and is daily ingested in quantities that far exceed an eventual use in electronics [36].

**Figure 1:**
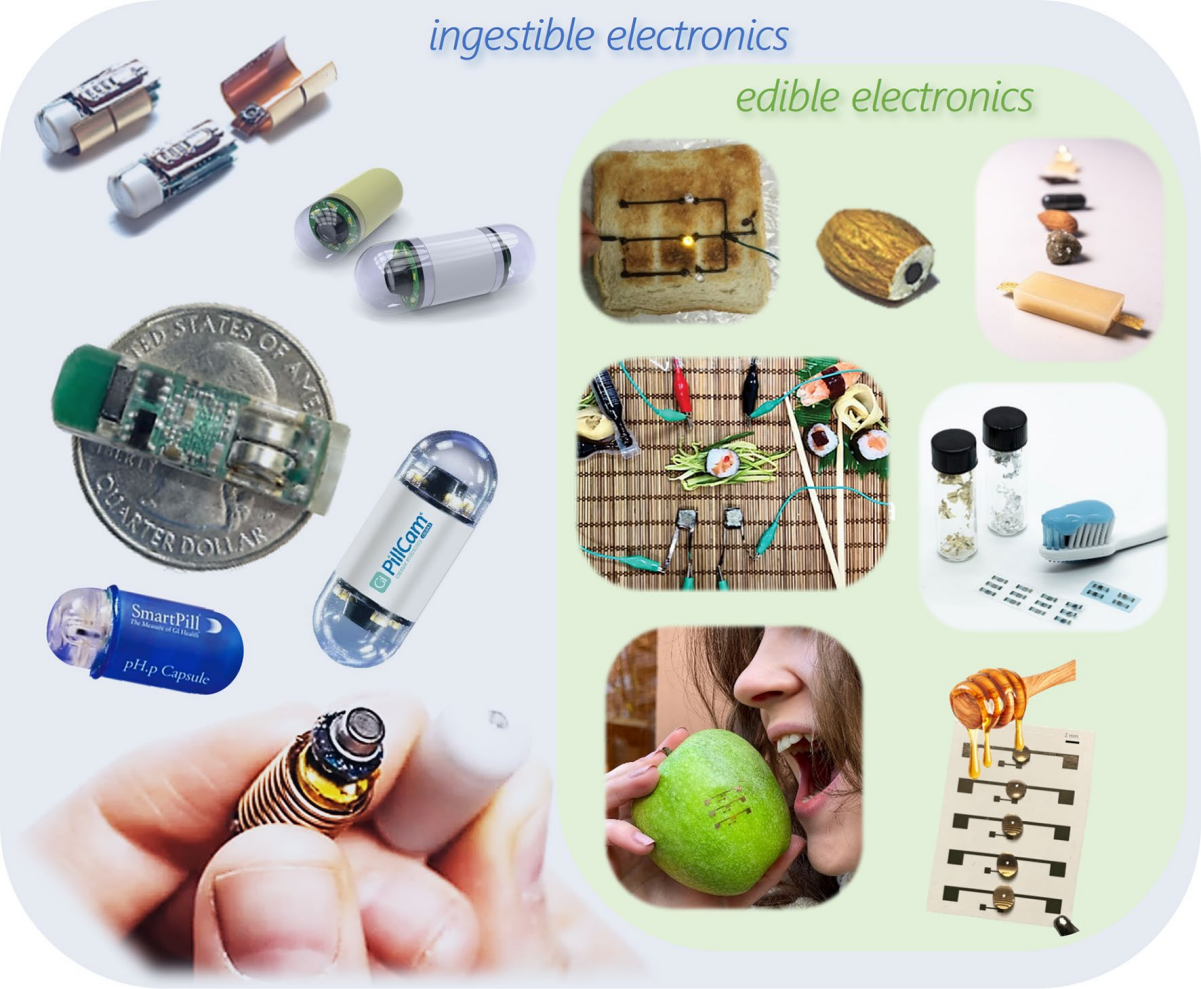
Overview of ingestible electronics technologies. The diagram illustrates the broader category of ingestible devices, including commercial smart pills such as SmartPill© and PillCam©, and research prototypes for medical diagnostics. A distinct subset of edible electronics is highlighted, featuring proof-of-principle demonstrators. While a complete fully edible pill system has yet to be developed, these advances are paving the way for both medical and non-medical applications, including food quality monitoring. Image reprinted (adapted) with permission from: Ref. [5], Copyright © 2018, Elsevier; Ref. [53], Copyright © 2017, Wiley; Ref. [54], Copyright © 2024. Wiley; Ref. [55], Copyright © 2021, Wiley.

Edible electronics can be therefore considered a subset of ingestible electronics (Fig. 1). Ingestible electronics comprises a wider set of electronic devices, typically in the form of inert pills encapsulating non-edible components, such as miniaturized PCBs, including silicon chips, physical and (bio)chemical sensors, light sources, and coin batteries [41]. Such devices are rigid and are not meant to degrade within the body, but to transit and being excreted unchanged after performing their function. Commercial devices comprise very powerful tools, allowing endoscopies [42], as well as more simple smart pills mostly limited to sensing pH [43], temperature, pressure, and gases [44, 45]. They have recently seen huge advancements in targeted monitoring of gut health thanks to two milestone reports: one demonstrates with animal testing the biochemical profiling of the GI tract with smart capsules that allow the simultaneous detection of electrolytes, metabolites, and hormones [46]; another one discloses a capsule, tested on humans, that allow to measure the redox balance along the gut [47]. Such devices provide extremely valuable information from the GI tract. Those at research level and some of the commercial ones are devised for administration in controlled hospital supervision or under specific conditions, while others have been suggested for self-administration without the need of recollection. However, inherent costs, risk of retention especially in an aging society, and concerns with increasing e-waste require attention [48–52].

Moving away from diagnostics, recurrent monitoring of biophysical markers to track, over an extended period, occurrence of abnormal fluctuations, would represent major advantages not addressable with current ingestibles. Examples would be systems designed for the monitoring of gut transit, gas production from microbial fermentation, and motility patterns: key physiological processes that are often altered in conditions such as diabetes, obesity, and irritable bowel syndrome. Continuously capturing these parameters could provide valuable insight into postprandial glycemic variability, metabolic efficiency, and symptom fluctuations, paving the way for more personalized, preventive strategies and reducing the need for hospital-based interventions. Devices serving such purpose should be easily accessible, ideally just by medical prescription, and compatible with self-administration. The latter requires technologies that can be safely ingested on a daily base, without requiring recollection and without raising environmental concerns. This scenario needs a different approach: a transition from simply ingestible, i.e., based on non-edible materials and encapsulated, to edible electronics, i.e., undergoing degradation within the body, either purely mechanical as fibers or metabolic, exploiting food electronic properties and/or incorporating non-toxic soft, typically carbon-based, electronic materials. Given the strong constraints and the immaturity of the field, edible electronics cannot rival ingestible electronics in terms of performances, and is proposed to offer alternative applications, trading-off complexity with safety and sustainability, in a fit-to-purpose and applications-oriented approach. We note that from an edibility perspective, the nutritional profile is not considered. Such aspect has been directly associated with nutritive electronics [15], which can be considered as a further subset of edible electronics and is relevant for materials present in largest volume fractions, such as insulators used as substrates and structural parts, for example, in smart pills.

## Research outputs in the field: a survey

**Figure 2:**
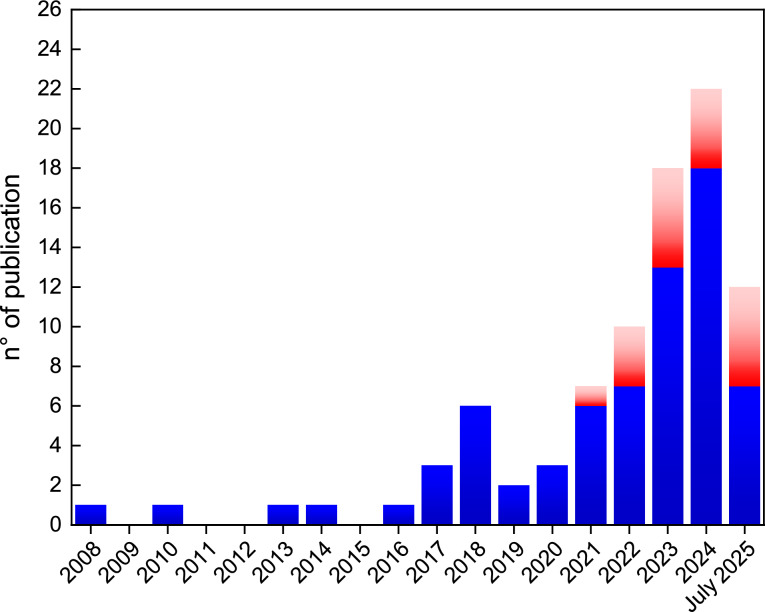
Number of indexed scientific papers making explicit reference to edible electronics up to July 2025. In red, the contributions from the ELFO project.

In Fig. 2 the number of scientific papers published every year up to July 2025 referring to edible electronics is reported [56]. Interestingly, the first report explicitly mentioning the expression “edible electronics” in an indexed journal is a short industry news article, dated 2008, written by Peter Harrop, founder of the technology consultancy IDTechEx, entitled “New Breakthroughs in Electronic Inks.” In this brief contribution, Harrop explores the potential impact and challenges of printed electronics in the near future. Within this context, and well ahead of practical research efforts, he envisages FDA-approved edible functional inks as the next game-changer, for the market of resorbable devices for food traceability and drug diagnostics that was emerging at that time [57, 58], but also for any application where accidental ingestion or direct contact with the human body may occur, such as electronic toys. Nevertheless, Fig. 2 clearly suggests that Harrop’s intuition of a high-impact scenario for edible electronics has remained largely unexplored by the scientific community until 2016, apart from isolated excursions of groups mainly involved in research on flexible, biodegradable, and green electronics, such as the works of the above-mentioned Irimia-Vladu and Bettinger. It is worth noting, however, that the scientific production up to 2016, though largely exploratory and fragmented, had already addressed critical aspects of the field, including powering, passive and active electronic building blocks, helping to establish many of the starting materials and strategic approaches that would later contribute to shape the field. Starting from 2016, the groups of Jiang, Wang, and in het Panhuis began explicitly referring to edible electronics as an independent research field and conducting research in this area with some continuity. From 2017 onward, the first demonstrators of functional edible components began to emerge, mostly proof-of-principle demonstrators still far from real-world applicability due to their large size and lack of integration. Nevertheless, they reflected a growing interest in articulating the practical relevance and potential impact of edible electronics, such as the non-invasive detection of physiologically relevant GI parameters (e.g., pH and pressure) and biomarkers. Year 2020, which corresponds to the starting of the ELFO project, marked a turning point in academic research on edible electronics, ushering in a new level of maturity. In the summer, the group led by Jiang at Arizona State University published “Edible and Nutritive Electronics: Materials, Fabrications, Components, and Applications” [15], a comprehensive and timely compendium of the progress that captured the momentum about to reshape edible electronics research. Soon after, in the fall, our review discussing the vision and the challenges of edible electronics appeared [16]. It is also interesting to note that in the same year, Onoe and co-workers presented at a conference the concept of a pill for intestinal bacteria monitoring made only of nontoxic materials for ingestion [59], a contribution that was published as a research paper only in 2022 [60]. This convergence of efforts reflects a broader, shared awareness of the field potential and the growing need for consolidation. Since then, a wide range of new functional materials and device architectures has emerged, leading to a steady increase of scientific output until 2024, strongly contributed by ELFO results, with a trend that appears respected also in 2025. These advances have begun to tackle longstanding challenges related to powering and communication systems, while also expanding the sets of sensing strategies and driving progress in edible integrated circuits, as will be detailed in the following section. Yet, it remains too early to assess whether this momentum will result in sustained scientific production: by considering the absolute numbers of published papers, the field is still a niche. Only a few players have consistently entered the field, such as Goran Stojanovic from the University of Novi Sad in Serbia and Kenta Fukada at the Shibaura Institute of Technology in Japan. The majority of other cases still appear to stem from isolated contributions by research groups primarily active in adjacent areas such as green and bioresorbable electronics.

## Towards edible electronic systems

**Figure 3:**
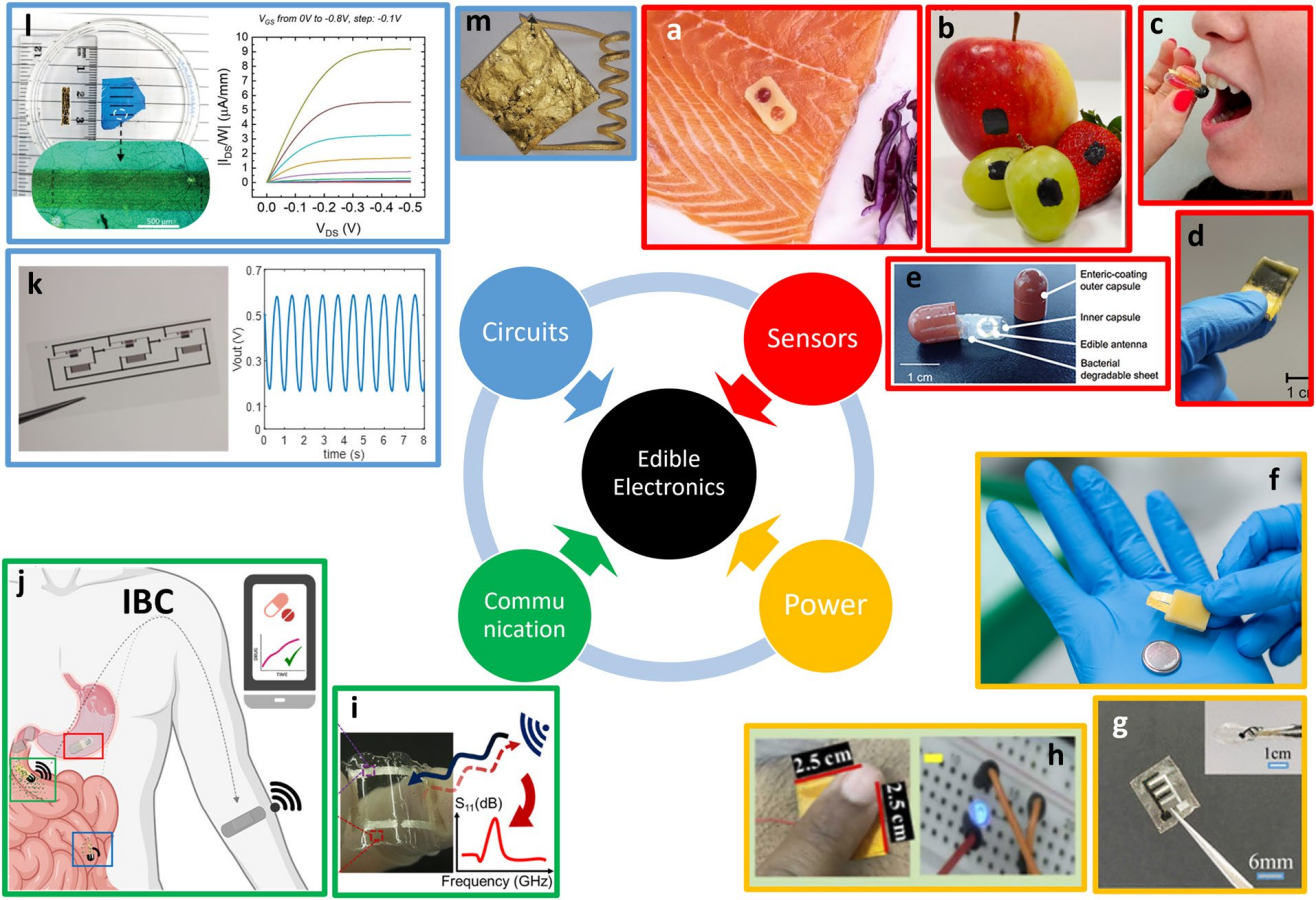
Components for future edible systems. *Sensors:* (a) defrosting sensor, realized with beeswax, edible metals Au and Mg, salted water, and red cabbage juice; adapted with permission from Ref. [61] Copyright © 2022, ACS. (b) Bioimpedance sensor realized with zein and activated carbon composite; adapted with permission from Ref. [28] Copyright © 2024, Wiley. (c) Tilt sensor realized in a gelatine 000 capsule with melt-mixed beeswax, sunflower oil, loaded with activated carbon, and edible gold foils; adapted with permission from Ref. [62] Copyright © 2023, Wiley. (d) Negative temperature coefficient resistive sensor, realized with activated carbon, gelatin candy, gold, ethyl cellulose, and beeswax, adapted from https://zenodo.org/records/13220770. (e) Gut bacteria sensor, realized in 000 gelatine capsule with gold, rice paper, and guar gum; adapted from http://www.onoe.mech.keio.ac.jp/research.html. *Power*: (f) rechargeable battery, realized with Riboflavin, Quercetin, edible gold, ethyl cellulose, activated carbon, nori algae, NaHSO_4_ in water, and beeswax; adapted with permission from Ref. [37] Copyright © 2023, Wiley. (g) Micro-supercapacitors, realized with activated carbon, Zinc, gelatin, and edible gold; adapted with permission from Ref. [64] Copyright © 2020, RSC. (h) Triboelectric nanogenerator, realized with chitosan and edible gold; adapted with permission from Ref. [65] Copyright © 2024, Elsevier.* Communication:* (i) thermally degradable split-ring resonators realized with edible silver leaves on gelatin substrates; adapted with permission from Ref. [66] Copyright © 2023, ACS. (j) Intra-Body Communication system, for the monitoring of Metformin release, based on the employment of activated carbon-based electrodes and a mixture of edible wax, edible oil, and hydroxyethyl cellulose to tune the drug release; adapted with permission from Ref. [31] Copyright © 2023, Wiley. *Circuits:* (k) ring oscillator realized with biocompatible semiconducting polymer (P3HT), ethyl cellulose, printed gold, and chitosan; adapted with permission from Ref. [40] Copyright © 2025, Wiley. (l) Electrochemical transistor, realized with CuPc, ethyl cellulose, printed gold, edible gold, and agar; adapted with permission from Ref. [26] Copyright © 2025, Wiley. (m) LC circuit realized with Italian spaghetti coated with edible gold leaves (solenoid), and seaweeds coated with edible gold foils (capacitor); adapted with permission from Ref. [18] Copyright © 2025, Wiley.

Any practical application of edible electronics will eventually require the development of a fully edible electronic system (Fig. 3). Systems typically require (i) sensors, to transduce target signals; (ii) analog and logic circuits, to condition sensors output and to perform computation; (iii) a communication pathway, to deliver data to the intended receiver, preferentially with a wireless approach; (iv) powering strategies, to provide the system with the required energy to operate. Here, we provide examples of recent developments in edible electronic components and of the first attempts of interconnection/integration. This is not meant to be a comprehensive account, but rather to represent some of the most significant progress towards systems from 2020. We would like to highlight here that various sensors and components, as well as edible functional materials, were proposed also before, as introduced in the first section and reported especially in the context of nutritive electronics [68].

### Edible sensors

Sensing comprises the greatest number of examples of edible electronic components. Recent proof-of-principle developments extended the potential target signals for the monitoring of the GI tract. Edible temperature sensors, made of activated carbon, gelatin candy, gold, ethyl cellulose, and beeswax, were reported for operation in the range from 5 to 50 °C [63]. Hydrogel capacitive sensors made of food materials were presented for redox reactions sensing [69]. Different pressure sensors were also proposed, including capacitive pressure sensors exploiting cellulose and pectin [70], and a resistive example made of a gelatin-based body, an activated carbon conductive coating, printed gold electrodes, and an ethyl cellulose substrate [71]. Moreover, a fully edible system has been proposed to wirelessly monitor gut bacterial activity by detecting the decomposition of a guar gum film [60]. For applications in environmental monitoring, edible sensors could favor the development of sustainable Internet-of-Things, without creating pollution and endangering wildlife: within such context, flexible humidity sensors were proposed, based on a K_2_CO_3_ and glycerol flour, operating from 6 to 94% relative humidity [72]. Among the sensors specifically developed for food monitoring, an edible defrosting sensor proposed by Ilic et al. [61] exploits a galvanic cell realized with edible metals, Au and Mg, and salted water. The latter work is one of the first to propose a possible system integration, with a proof-of-principle interconnection to an optical readout, a Sn–Au cell exploiting the ionochromic properties of anthocyanins present in red cabbage juice to permanently mark a defrosting event. In the same field, an edible conducting glue, based on zein and activated carbon composites, was recently exploited for stable bioimpedance sensors enabling post-harvesting monitoring of fruit aging [28]. Gelatin and AC were adopted to realize an edible conductive composite that enabled multimodal sensing, including real-time motion tracking, respiration monitoring, speech recognition, and contactless thermal sensing [73]. Edible electronics may also find adoption in future edible robotics (see the recent review [74] for an overview of this specific field). In this case, an edible piezoresistive strain sensor, exploiting electronic conduction, rather than ionic conductivity at the base of conductive hydrogels [6], was presented [75]. Additionally, to provide edible robots with perception of orientation, a simple but robust bistable and resistive tilt sensor was demonstrated, starting from a known non-edible bistable tilt sensor architecture, and replacing each material with edible ones [62].

### Edible circuits

Circuits require both passive and active components. Edible passives were among first components to be proposed [68], and have recently seen a development towards integrated microelectronics, with inkjet printed resistors of nano-activated carbon formulations, delivering a resistivity of 6.6 Ω cm and a sheet resistance tunable with the number of printing passes [76]; yet, the nanometric size of the carbon, different from the micrometric one approved for edibility, will require thorough toxicological studies. Various edible capacitors have been reported [77], including lateral architectures exploiting dielectrics obtained from recycled food waste, such as lemon, orange, grapefruits, apple, and banana, among others [38], displaying pF capacitance. Typically, such works are associated also to the realization of edible inductors [24], with recent examples reporting solenoids obtained by coating spaghetti with edible gold leaves, with inductances in the range of 100 nH [18], and silver-coated isomalt coils achieving µH range [67]. Active components are instead far less developed, although more recent works dedicated to demonstrating low-voltage electrolyte-gated transistors appeared. The use of hydrated honey to drive complementary organic transistors [55] was recently suggested, followed by the use of a chitosan:glycerol formulation to gate transistors based on evaporated CuPc semiconductor, in a fully edible architecture [36]. CuPc, a well-known organic semiconductor, is used as a blue pigment and whitening agent in commercial toothpastes. Its unavoidable ingestion, in the order of 1 mg/day, has not led to any adverse reaction, indicating it as a relevant candidate for edible electronics. Direct extraction from toothpaste was also demonstrated, leading to functional, solution-processed devices [26]. Printed and potentially edible transistors, based on the same device architecture but exploiting a non-cytotoxic polymer semiconductor known as P3HT, were also proposed [35]. Despite the absence of a clear toxicological profile for P3HT, which is present in small ng traces per device, this platform was the first one adopted to demonstrate integrated logic circuits, in the form of NAND gates, a ring oscillator and a latch [40].

### Communication

Any edible pill will have to communicate data to the outside of the body, and any food tag will have to be interrogated. To fill this gap and to enable a radio-frequency (RF) wireless link, a series of LC resonators with resonant frequencies in the range from 10 to 100 s of MHz were realized by interconnecting some of the edible components presented above [38, 69]. Additionally, thermally degradable split-ring resonators realized with edible silver leaves on gelatin substrates operating in the Ultra-High Frequency (UHF) range were also proposed [66], along with split-ring resonators made from rice paper and gold and operating in the GHz range [60]. Alternatively, in order to cope with the limitations of edible electronics and offer more accessible, lighter and more energy-efficient sensing and communication modules with respect to radio front-ends, the use of Intra-Body Communication (IBC), also known as Human Body Communication (HBC) [78], was proposed. In a recent report, the possibility to modulate the amplitude of a synthetic signal coming from an edible pill was demonstrated with an *in-vitro* simulation and exploited to monitor passive drug release, namely Metformin [31].

### Power

Besides passives solutions that could cover the needs of some applications, edible energy storage devices are required to increase the available power at the node, the communication range and bandwidth, as well as continuous monitoring over time. Recent examples see the predominance of edible supercapacitors. A gravimetric energy density of 3.36 mW h g^−1^ and a gravimetric capacity of ~ 9 mAh g^−1^, with a stability exceeding 1000 charge–discharge cycles, was achieved in edible supercapacitors exploiting electrodes based on activated carbon and ethyl cellulose composites [79]. The electrodes showed also triboelectric properties when adopted as electropositive elements in organic triboelectric nanogenerators. Additionally, a food-based triboelectric nanogenerator, made of chitosan and edible gold, was recently proposed for electronic skin applications [65]. Edible supercapacitors exploiting zwitterionic and edible gel electrolytes based on hydroxyethyl cellulose and commercial soy sauce (shoyu) were shown to display a maximum specific capacitance of 3.75 F g^−1^, with a retention of 86.5% after 10 000 cycles [54]. Also micro-supercapacitors have been recently proposed, with examples including a microdevice tested both *in-vitro* and *in-vivo*, delivering high energy density of 0.22 mWh cm^–2^ and a high working voltage of 1.8 V, made with an activated carbon microcathode and zinc microanode, fitting standard capsules [64]. Similarly, micro-supercapacitors made of food, achieving capacity of 10.86 μW h cm^−2^ and power density of 0.78 mW cm^−2^, could be coiled into a medical endoscopy capsule and operated in simulated gastric fluids [23]. Besides supercapacitors, also edible batteries saw a tangible progress, with the demonstration of the first edible rechargeable redox battery, showing an output voltage of 0.65 V and a capacity of about 10 µAh based on riboflavin for the anode and quercetin for the cathode [29]. The electrodes were realized with a composite of activated carbon and ethyl cellulose, coated on edible gold leaves acting as collectors, while the used separator is Nori, soaked with a NaHSO_4_ water electrolyte.

### Integration

**Figure 4:**
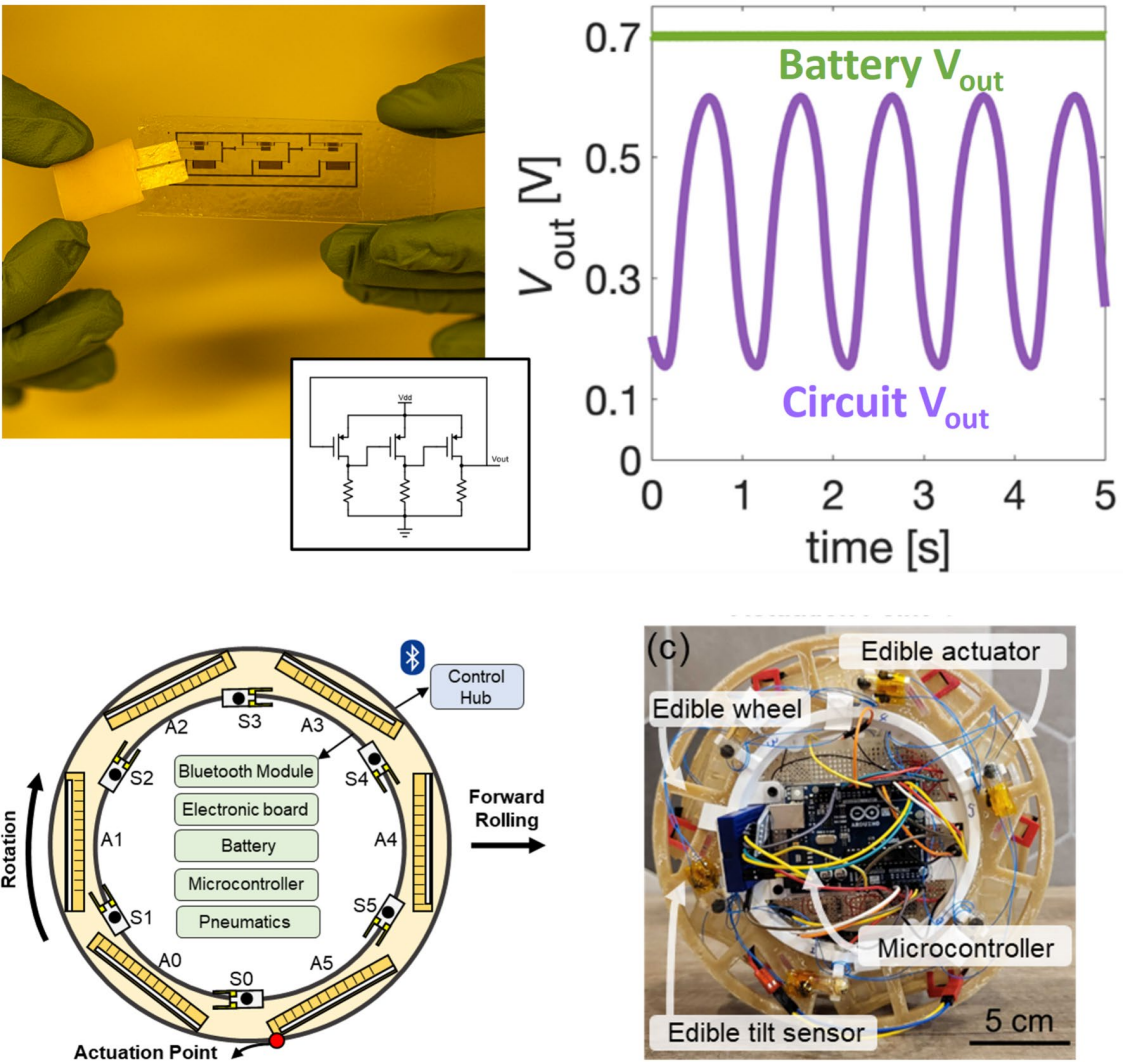
Some of the first examples of integration of edible components. Top: a potentially edible ring oscillator, mostly based on edible materials with nanogram traces of a biocompatible, non-cytotoxic polymer semiconductor, powered by an edible rechargeable battery adapted with permission from Ref. [40] Copyright © 2025, Wiley. Bottom: a partially edible rolling robot, integrating an array of 6 tilt sensors to provide perception of orientation. Adapted with permission from Ref. [62] Copyright © 2023, Wiley.

Despite its simplicity, the above-mentioned sensor for gut bacteria represents the only demonstration for a fully edible, fully integrated in-capsule system for gut monitoring [60]. In fact, examples of integration are extremely rare and typically limited to isolated subcomponents. First examples were described above for simple resonating LC circuits and printed integrated logic circuits with a scale of integration limited to few transistors. Recently, a coplanar version of the edible battery was proposed to favor integration with other components, displaying an increased capacity of 20 µAh thanks to a higher mass loading [37]. As reported in Fig. 4, the possibility to power potentially edible logic circuits directly with the coplanar edible battery was demonstrated thanks to the matching battery output voltage and transistor operation voltage [40]. Towards the integration of edible electronics with edible actuators, a resistive strain sensor was directly spray coated on a gelatin actuator and powered by the same edible battery, producing a proof-of-principle sensorized edible gripper [75]. Additionally, an array of 6 edible tilt sensors were mounted on a partially edible rolling robot, made with an edible oleogel composite wheel and edible gelatin actuators [62]. The latter allowed the robot to operate in a closed-loop, perceiving orientation from the sensors in order to trigger the correct actuator.

## Perspectives and major challenges

From this brief survey over the last few years, a marked progress in the field of edible electronics emerges. Many of the missing components required for future edible smart pills and electronic tags have been addressed and developed at a proof-of-principle level. Nevertheless, realizing the full potential of genuinely edible technologies for health and food safety monitoring will require significant further progress and the resolution of several remaining challenges. In the following, we provide a critical view on the most significant ones to be addressed in relation to edible functional materials, components, manufacturing, and regulations. Fields of application where edible electronics could be credibly exploited, beyond ingestible smart pills and food tagging, are then discussed.

### Functional materials

A major constraint arises from the edibility requirement of the functional materials to be adopted, which, when combined with other critical needs such as processability and mechanical robustness, drastically limits the set of available candidates. This applies particularly to materials exhibiting key electronic functions, such as conductors forming device electrodes and semiconductors responsible for the operation of active electronic components. Carbon-based conductors that are edible in large doses (mg to g per kg of body weight) are quite resistive, exceeding few Ω/cm, because of the unfavorable form factor, as particles in powders are micron sized. On the other hand, edible metals, such as gold and silver, are only allowed in small amounts (µg per kg of body weight), with their edibility approved so far only in the case of specific physical formats, such as foils or flakes, raising concerns about the safety of other form factors, including metallic nanometric patterns, especially those obtained from metallic inks. As for semiconductors, so far only cosmetic pigments used in toothpastes and known biocompatible polymer semiconductors offer reasonable performances. Nevertheless, the actual adoption of known or newly designed biocompatible semiconducting polymers would still require stringent, costly, and time-consuming testing to demonstrate a toxicological profile compatible with safe ingestion. Such regulatory effort is unlikely to raise significant concerns, as the semiconductor can perform effectively even when present only in imperceptible traces, i.e., nanograms per device, but it would still require considerable time, resources, and clear market justification. In contrast, the cosmetic pigments, though not yet approved for ingestion, are supported by relevant toxicity data and could therefore follow a smoother path towards applications. Still, their use would require formal validation by the appropriate regulatory agencies. In Europe, this process typically takes an average of about 2.5 years, including EFSA’s scientific assessment and subsequent EU-level authorization [80]. In the United States, the approval of a new food additive through the FDA’s petition process can take significantly longer depending on the completeness of the dossier and the need for additional data [81]. Aiming at an edibility by design also of the semiconductor, it is quite striking that, despite their early identification, there is a lack of convincing devices exploiting inherently edible or even nutritive semiconductors. Carotenoids, for example, were only explored years ago and showed limited performance, a result that may have led to premature dismissal [19]. However, considering the largely improved understanding of structure–function relationships in organic semiconductors, these materials may well deserve renewed attention. A thorough re-investigation should clearly not only reassess their electronic performance, but also address issues related to stability and, not less importantly, evaluate the impact of residual impurities resulting from extraction processes, particularly when sourcing from bio-based materials such as food waste.

### Components

Beyond functional edible materials, the progress in the last few years mostly stops at devices and components level. Already at component level, several important building blocks are missing. Among active devices, there is an obvious gap in diodes and rectifiers, as well as amplifiers and analog circuits, required to condition the sensors output. Moreover, initial demonstrations of transistors and simple logic gates based on plausibly edible semiconductors all rely on electrolyte-gated architectures to enable low-voltage operation, an approach further supported by the broad availability of edible electrolytes. However, this choice introduces application-level limitations, including inherently slow ion transport within the electrolyte and stringent circuit design constraints that hinder the minimization of parasitism. These challenges will need to be addressed in future developments, depending on the minimum requirements, for example in terms of speed or bandwidth, of each specific application.

Further research opportunities can be identified. Despite the pioneering work on edible electrochemical sensors by Wang and collaborators [53], edible biosensors, capable of detecting specific markers in the GI tract, are still largely missing [82]. With the perspective of embedding monitoring capabilities in edible pills, edible biosensors may represent the next avenue of progress in the field. At the same time, the electrolyte gating of organic semiconductors preferentially adopted for transistors in this field is currently under intense exploration for neuromorphic applications [83]. With the perspective to develop an efficient, low-power, and lower complexity electronics, we believe that also edible neuromorphic offers ample opportunities. To this aim, a first pioneering example exploiting liquid leg albumen for reservoir computing has been just published [84].

### Manufacturing and regulations

With the goal of enabling future edible systems, a critical limit lies in the proof-of-principle nature of most of the proposed components, making them hardly compliant with a future system integration. Mostly, they have been devised to prove a functionality, but aspects such as form factors and manufacturability have not been convincingly addressed. One has also to consider that interconnections of fragile components, not robust to high temperatures, will eventually require the development of specific processing strategies. Such aspects, together with reliability, shelf-life, and operational stability, pose severe risks for turning edible electronics in a concrete opportunity if not properly assessed and investigated. We expect that, if sufficiently reliable systems can be eventually obtained, they will follow a fit-for-use approach, strongly application driven. In the longer term, further obstacles for edible electronics will be the approval for ingestion at system level, as the use of inherently edible materials will not suffice [17]. The absence of standards will also have to be pragmatically addressed before any real product can be envisaged. An almost complete absence of *in-vivo* studies and of studies dedicated to the acceptance by the general public represent additional gaps.

### Emerging application fields

While challenges and risks are still enormous, the field appears to be justified by an increasing need for ingestibles [85, 86] that can solve retention hazard and not contribute to an increase of e-waste. Interestingly, there are emerging applications that can only be addressed with electronics that are truly edible. The recently conceived field of edible robotics [74] provides a platform where edible electronics would be key in embedding sensing, computation, and control in different contexts, from edible robots for feeding endangered persons, wildlife, and farmed fish, to robotic food, offering fascinating culinary experiences and new tools to treat eating disorders. We also expect that key edible components may contribute to the development of sustainable electronics at large, as in the case of edible batteries that could be used to drastically reduce the environmental impact of Internet-of-Things, especially in the context of deployment of a large number of nodes over large areas, as required for example in AgriTech [37].

In summary, edible electronics is experiencing a progress both in terms of materials, processes, and components, with the involvement of a limited but increasing number of research groups. It is now therefore easier to envision a wide set of potential applications and systems starting from current achievements. Among those, the potential for biomedical monitoring applications, as opposed to diagnostic ones, has valid motivations. Yet, critical aspects have only started to be addressed or are yet to be convincingly addressed. The field can move forward only by addressing the current stringent limits with a multidisciplinary approach, with complementary competences from diverse fields as materials and food science, electronics and bioelectronics, nutrition and medicine, including physicians from different branches, such as grastroenterologists. Edible electronics will greatly benefit if more research groups and scientific communities join the development of its appealing scenarios.
